# Ciclesonide exhibits lung-protective effects in neonatal rats exposed to intra-amniotic enterotoxin

**DOI:** 10.3389/fped.2024.1428520

**Published:** 2024-09-24

**Authors:** Victoria Mielgo, Elena Gastiasoro, Chiara Catozzi, Francesca Ricci, Miguel A. Gomez-Solaetxe, Xabier Murgia, Carmen Rey-Santano

**Affiliations:** ^1^ Animal Research Unit, Biocruces-Bizkaia Health Research Institute, Barakaldo, Spain; ^2^ Primary Health Care, Biocruces-Bizkaia Health Research Institute, Barakaldo, Spain; ^3^ R&D Department, Chiesi Farmaceutici, Parma, Italy; ^4^ Medical Devices Group, University of the Basque Country (EHU), Portugalete, Spain; ^5^ Scientific Consultant, Bilbao, Spain

**Keywords:** ciclesonide, corticosteroids, brain, bronchopulmonary dysplasia, enterotoxin

## Abstract

**Introduction:**

Despite the advances in perinatal care, bronchopulmonary dysplasia (BPD) continues to be a highly prevalent chronic lung disease that affects newborns, especially affecting premature newborns. There is no specific cure for BPD, and treatments aimed at reducing the risk of developing BPD focus mainly on lung-protective ventilation strategies, surfactant therapy, and/or corticosteroid administration. Our objective was to evaluate whether systemic postnatal administration of a new glucocorticoid, ciclesonide, can attenuate the alteration of lung structure and pulmonary hypertension in a rat model of chorioamnionitis-induced BPD, with minimal adverse effects on the developing brain.

**Methods:**

Endotoxin (ETX) or saline was administered to pregnant rats by intra-amniotic (i.a.) injection on day 20 of pregnancy, and pups were delivered by cesarean section on day 22. Ciclesonide (0.5 mg/kg) was administered postnatally for five consecutive days to pups previously exposed to i.a. ETX. On postnatal day 14, we assessed lung function (compliance), lung structure (radial alveolar count, mean linear intercept, pulmonary vessel density), pulmonary hypertension, and brain histology (edema, inflammation, apoptosis, hemorrhage, and infarction).

**Result:**

On postnatal day 14, the effects of i.a. ETX administration were evident in neonatal rats not receiving treatment; these animals showed impaired lung compliance, disrupted lung structure, and developing pulmonary hypertension compared to those receiving i.a. saline. Postnatal administration of ciclesonide for 5 days was associated with significantly better outcomes in terms of lung compliance, alveolarization, lung vascular growth, and pulmonary hypertension, without affecting the brain histological parameters evaluated.

**Conclusion:**

Postnatal ciclesonide administration preserved lung function and structure and prevented pulmonary hypertension in a BPD model induced by antenatal i.a. ETX administration, without causing any adverse effects on brain development. These findings suggest that the new glucocorticoid, ciclesonide, may provide a novel strategy for the prevention of BPD; however, more long-term studies are required.

## 1 Introduction

Bronchopulmonary dysplasia (BPD) is one of the main chronic lung diseases in infancy. It is related to premature birth and injury to the immature lung that occurs during antenatal and early postnatal life and continues to be one of the most serious sequelae of prematurity. Despite advances in perinatal care over recent years (1, 2), the incidence of BPD has remained high and has not significantly improved (3, 4). Therefore, it is imperative to continue investigating the mechanism of BPD pathogenesis as well as new approaches to its treatment.

Many pre- and postnatal factors have been described as possible causes or aggravators of BPD, including chorioamnionitis, placental dysfunction, ventilator-induced injury, hyperoxia, infection, and inflammation (5, 6). Research into the pathophysiology of BPD has provided growing evidence of the importance of the relationship between BPD and inflammation (7–9). The use of corticoids (potent anti-inflammatory drugs) has been proposed as a treatment to reduce the risk or severity of BPD (10–12). Nonetheless, the administration of the most widely used postnatal corticosteroids (mainly dexamethasone and hydrocortisone) is not free of adverse effects, including intestinal perforation, growth failure, arterial hypertension, and a negative impact on the developing brain (12, 13).

Ciclesonide is an inhaled glucocorticoid used to treat asthma and allergic rhinitis. This new synthetic glucocorticoid has also been proposed as a potential candidate to prevent or treat BPD. It can be administered systematically and has shown selective activation of the glucocorticoid receptor in the neonatal lung and limited adverse neurodevelopmental effects (14) in a neonatal rat model. Previous studies have shown that antenatal injection of endotoxin (ETX) to induce intrauterine inflammation, as observed in chorioamnionitis (15, 16), induces fetal pulmonary hypertension and lung structure abnormalities, which mimic features of human BPD (17, 18). This background led us to hypothesize that ciclesonide would attenuate the alteration of lung structure and pulmonary hypertension in a rat BPD model of chorioamnionitis and, unlike the most commonly used corticoids, would offer these benefits with negligible adverse effects on the developing brain.

## 2 Materials and methods

The experimental protocol complies with European and Spanish regulations for the protection of experimental animals (UE2010/63 and RD53/2013) and has been approved by the Ethics Committee for Animal Welfare of the Biobizkaia Health Research Institute (OEBA-CET-2021-012).

### 2.1 Study design

#### 2.1.1 Intra-amniotic enterotoxin administration

The procedures were adapted from Tang et al. (17). In brief, pregnant rats were given intra-amniotic (i.a.) injections on day 20 day of pregnancy (vs. term at 22 days). This injection timing was chosen because it corresponds to the late canalicular stage of lung development in rats, and at this point, rats show a similar stage of lung development to that in human premature newborns born at 24–26 weeks of pregnancy, who are at very high risk of developing BPD. After premedication with 0.01–0.05 mg/kg of buprenorphine injected subcutaneously, laparotomy was performed on the pregnant rats under general anesthesia (induced with sevoflurane 3%–5% via a face mask; Surgivet, St. Paul, MS, USA). Throughout these procedures, the animals were kept on heating pads to prevent hypothermia (HB101/2, Panlab, Barcelona, Spain).

Pregnant rats were randomized into control (CTL) or ETX groups: the CTL group received 50 μl of saline solution per amniotic sac, while both ETX groups (treated and untreated; see below) received 10 μg of *Escherichia coli* O55:B55 ETX (Sigma Chemical, St. Louis, MO, USA) diluted to 50 μl with saline solution per amniotic sac. Under sterile conditions, a 3–4-cm-long midline abdominal incision was made to expose the amniotic sacs for i.a. injections, and up to a maximum of 8 sacs per dam were identified and injected with saline or ETX. This limit (8 sacs/dam) was set to reduce the risk of maternal death due to systemic toxicities from high cumulative doses of i.a. ETX.

Abdominal incisions were closed, and incision wounds were irrigated with bupivacaine (1–2 mg/kg injected intramuscularly) for pain control. Pregnant rats were closely monitored to confirm that they regained consciousness within 10 min after the surgery and subsequently, placed back into cages and observed for activity, feeding and drinking patterns, and signs of bleeding or infection.

#### 2.1.2 Cesarean section

On day 2 after i.a. ETX injections, the rat pups were delivered by cesarean sections under general anesthesia (induced as described above). Cesarean delivery was performed rather than allowing vaginal delivery to identify the fetuses exposed to i.a. ETX or saline, based on the order of the sacs and their anatomic locations with respect to the ovaries. The fetuses in the amniotic sacs that had received injections were identified and delivered, all within 5 min of anesthesia induction, and the dams were then euthanized with intraperitoneal pentobarbital sodium. Immediately after birth, the pups were placed on heating pads and dried manually with gauze sponges; within 30 min, they were placed with foster mother rats in standard cages.

#### 2.1.3 Groups

- Control group (CTL, *n* = 23): Pregnant rats received an i.a. injection of normal saline, and the neonatal pups did not receive any treatment.
- Enterotoxin group (ETX, *n* = 21): Pregnant rats received an i.a. injection of 10 μg of *E. coli* O55:B55 ETX (Sigma Chemicals, St. Louis, MO, USA) diluted to 50 μl with normal saline per sac, and the neonatal pups did not receive any prophylactic treatment.
- Enterotoxin plus ciclesonide group (ETX-Cicle, *n* = 20): Pregnant rats received an i.a. injection of 10 μg of *E. coli* O55:B55 ETX diluted to 50 μl with normal saline per sac, and the neonatal pups received subcutaneous injection of ciclesonide (0.5 mg/kg, SML1955, Sigma-Aldrich, St. Louis, MO, USA) at the nape of the neck (10 μl/g) once a day for five consecutive days [from postnatal day 1 (PN1) to postnatal day 5 (PN5)] (14).

### 2.2 Study measurements

#### 2.2.1 Survival rate and body weight

Survival rate was recorded at birth and on P14. Body weight was measured within 30 min after birth, on postnatal day 7, and just before being sacrificed for study measurements.

#### 2.2.2 Lung mechanics

On postnatal day 14, neonatal pups were anesthetized, and a catheter was placed in their trachea and connected to a small animal ventilator (VentElite, Harvard Apparatus, Holliston, MA, USA) with the following settings: tidal volume of 6 ml/kg, positive end-expiratory pressure of 3 cmH_2_O, and a respiratory rate of 90 bpm. The peak inspiratory pressure (PIP) reached by each neonatal pup under the preset ventilation settings was measured, and lung compliance was calculated as follows: tidal volume/[(PIP − positive end-expiratory pressure) × body weight].

#### 2.2.3 Lung tissue processing and analysis

After lung mechanics measurements, the pups were sacrificed using pentobarbital, as was done for the mother rats. For fixation, the lungs were inflated with 4% formaldehyde administered through a Luer lock cannula inserted into the throat of each pup and maintained at 15 cmH_2_O pressure for 60 min. A ligature was tightened around the trachea to maintain the pressure, and the cannula was removed. The lungs were then dissected and immersed in 4% formaldehyde at room temperature overnight. Subsequently, 2-mm-thick transverse slices were cut from lung samples and embedded in paraffin. Lung immunohistochemical and morphometric analyses were carried out by pathologists blinded to group allocation as follows.

Five-micrometer sections were taken, mounted on slides, and stained with hematoxylin and eosin for the assessment of alveolar structure by morphometric analysis [radial alveolar count (RAC) and mean linear intercept (MLI)]; additionally, von Willebrand Factor (vWF), an endothelial cell-specific marker, was used to assess pulmonary vessel density (PVD) and pulmonary vessel wall thickness (PVWT). In each pup, we obtained at least five readings for RAC and measured at least 10 pulmonary vessels for PVD and PVWT.

RAC: Alveolarization was assessed using standard RAC methods developed by Emery and Mithal (19, 20). Respiratory bronchioles were identified as bronchioles lined by epithelium in one part of the wall. We counted the number of septae intersected by a perpendicular line drawn from the center of the respiratory bronchiole to the edge of the connective tissue, septum or pleura, surrounding the acinus.

MLI: To calculate the MLI, we superimposed a grid on the image, counted the number of times the alveolar walls were intercepted by the grid lines, and then used the following formula: MLI = (*N*)(*L*)/*m*, where *N* is the number of superimposed lines, *L* is the length of the superimposed lines, and *m* is the number of times the alveolar walls are intercepted by the grid lines.

PVD: PVD was determined by counting vWF-stained vessels with an external diameter of less than 100 μm per high-power field. Fields containing large airways or vessels were excluded from the analysis.

PVWT: Wall thickness and external diameter were measured in pulmonary vessels with an external diameter of 10–30 μm using Adobe Photoshop CS (San Jose, CA, USA). The percent medial thickness of an individual vessel was then calculated using the following formula: (medial thickness × 2 × 100)/external diameter.

#### 2.2.4 Right ventricular hypertrophy measurements

The heart was isolated, and the right ventricle (RV) and left ventricle plus septum (LV + S) were dissected and weighed. We then calculated the ratio of RV to LV + S weights to assess right ventricular hypertrophy.

#### 2.2.5 Brain tissue processing and analysis

Brain samples were obtained, immersed in 4% formaldehyde at room temperature, and subsequently embedded in paraffin. Histological evaluation studies were carried out by pathologists blinded to the study, assessing necrosis, edema, inflammation, hemorrhage, and infarction (21). Briefly, 20 fields were analyzed. Pathological features in the brain (inflammation, hemorrhage, and edema) were each scored on a 0–3-point scale: 0 corresponded to no injury, while 1, 2, and 3 indicated mild injury, moderate injury, and severe injury across the field, respectively. The presence of more than five necrotic cells per field was considered indicative of neuronal necrosis (score range: 0–20), while the presence or absence of infarction was scored as 1 or 0, respectively.

More specifically, inflammation and hemorrhage were assessed in each brain section by measuring the number of perivascular inflammatory foci in the case of inflammation and the number of microscopic hemorrhagic foci in the case of hemorrhage. Summing over the 20 fields analyzed, these values were scored as follows: 0 corresponded to an absence of foci, while 1, 2, and 3 indicated the presence of less than 4 foci, 4–10 foci, and more than 10 foci, respectively.

### 2.3 Data analysis

Values were expressed as mean ± standard deviation (SD). Intragroup comparisons were made using a one-factor analysis of variance (JMP8, Statistical Discovery, SAS, NC, USA). Analyses were performed with the Bonferroni–Dunn correction. A *p*-value <0.05 was considered significant.

## 3 Results

### 3.1 Survival rate and body weight

After receiving i.a. ETX, 75% of rat pups were alive at birth compared to 100% of those receiving i.a. saline (CTL group). By P14, 100% of neonatal rats were alive in the CTL and ETX-Cicle groups, while survival was 80% in the ETX group.

At birth, pups exposed to antenatal endotoxin (ETX and ETX-Cicle groups) had significantly lower body weight than those exposed to saline injection (CTL group) (Table 1). Nonetheless, by postnatal days 7 and 14 (P7 and P14, respectively), all studied groups showed similar body weights, with the between-group differences not reaching significance (Table 1).

**Table 1:** Effect of ciclesonide administration on body, heart, lung, and brain weights in an experimental i.a. enterotoxin administration model.

Group	F:M ratio	Body weight (g)			Heart weight (g)	Lung weight (g)	Brain weight (g)
		P0	P7	P14			
CTL	12:11	6.6 ± 1.0*	16 ± 3	29 ± 5	0.19 ± 0.05	1.4 ± 0.6	1.2 ± 0.1
ETX	9:12	5.4 ± 0.8	14 ± 3	27 ± 7	0.19 ± 0.06	1.5 ± 0.6	1.2 ± 0.1
ETX-Cicle	9:11	5.3 ± 0.5**	15 ± 2	30 ± 5	0.21 ± 0.05	1.7 ± 0.5	1.3 ± 0.1

F:M ratio: female:male ratio; P0: postnatal day 0; P7: postnatal day 7; and P14: postnatal day 14.

The numbers of pups studied in each group are as follows: control (CTL), *n* = 23; enterotoxin (ETX), *n* = 21; ETX-ciclesonide (ETX-Cicle), *n* = 20.

* *p* < 0.05 vs. ETX group; **p < 0.05 vs. CTL group.

### 3.2 Lung mechanics

**Figure 1:**
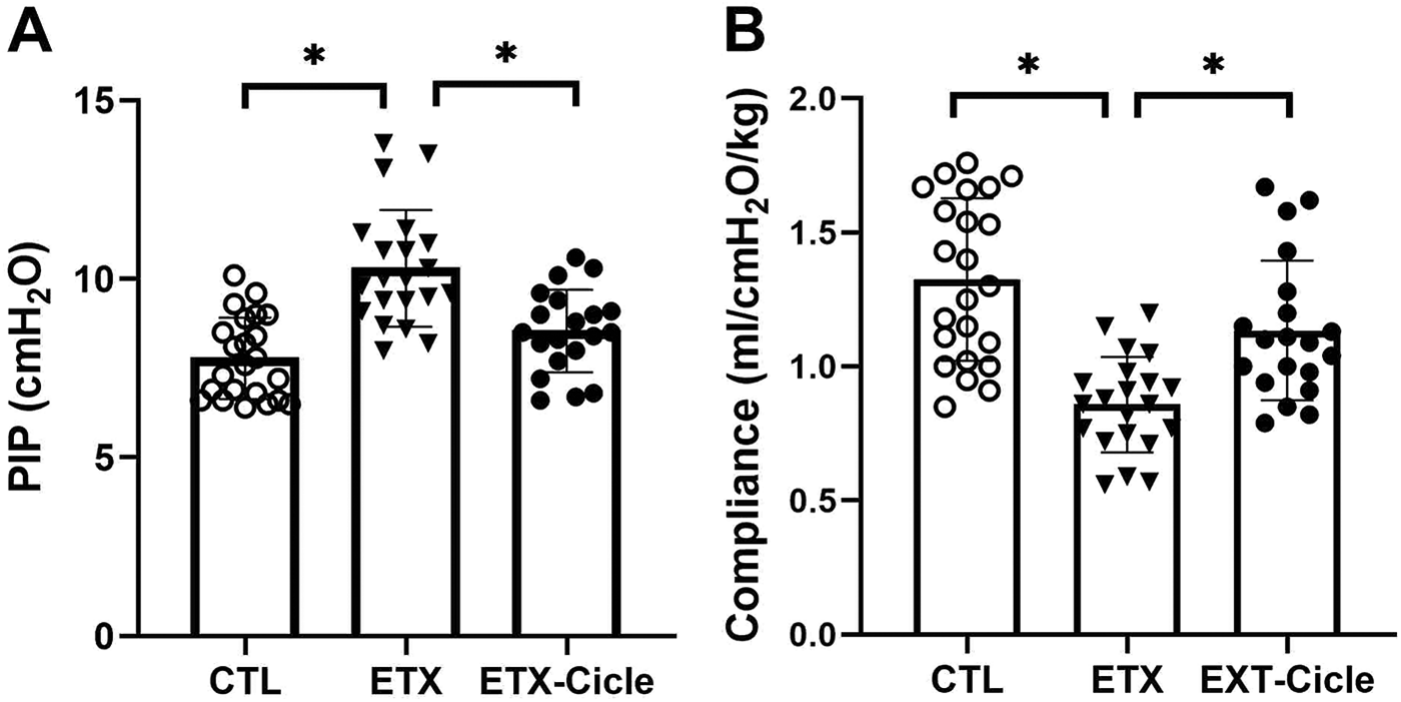
Effect of ciclesonide administration on lung function in an experimental i.a. enterotoxin administration model. Values of **(A)** PIP and **(B)** lung compliance in saline-control rats (CTL), rats who received i.a. exposure to enterotoxin (ETX), and rats who received i.a. exposure to enterotoxin and subcutaneous administration of ciclesonide (ETX-Cicle). The numbers of pups studied in each group are as follows: CTL, *n* = 23; ETX, *n* = 21; ETX-Cicle, *n* = 20. *p < 0.05 vs. ETX group.

At P14, significantly higher PIP values with significantly lower compliance values were observed in the ETX group than in the CTL group (Figures 1A, B). Meanwhile, PIP and compliance in rats exposed to i.a. enterotoxin that received ciclesonide treatment (ETX-Cicle) reached values similar to those in the CTL group.

### 3.3 Lung histological morphometric analysis

**Figure 2:**
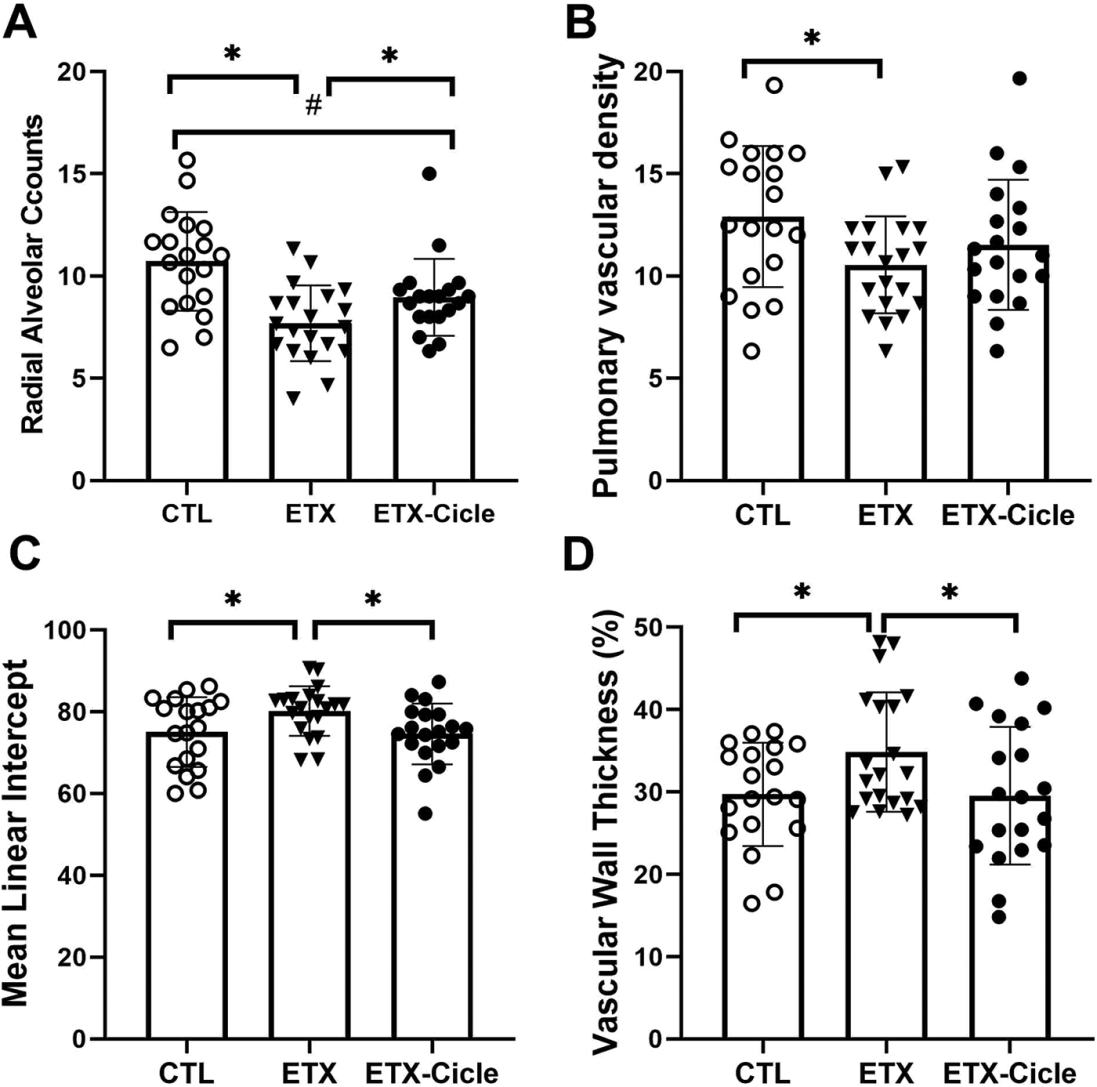
Effect of ciclesonide administration on lung morphometric analysis in an experimental i.a. enterotoxin administration model. Values of **(A)** radial alveolar counts, **(B)** mean lineal intercept, **(C)** pulmonary vascular density, and **(D)** pulmonary vascular wall thickness in saline-control rats (CTL), rats who received i.a. exposure to enterotoxin (ETX), and rats who received i.a. exposure to enterotoxin and subcutaneous administration of ciclesonide (ETX-Cicle). The numbers of pups studied in each group are as follows: CTL, *n* = 19; ETX, *n* = 20; ETX-Cicle, *n* = 19. *p < 0.05 vs. ETX group; #*p* < 0.05 vs. CTL group.

**Figure 3:**
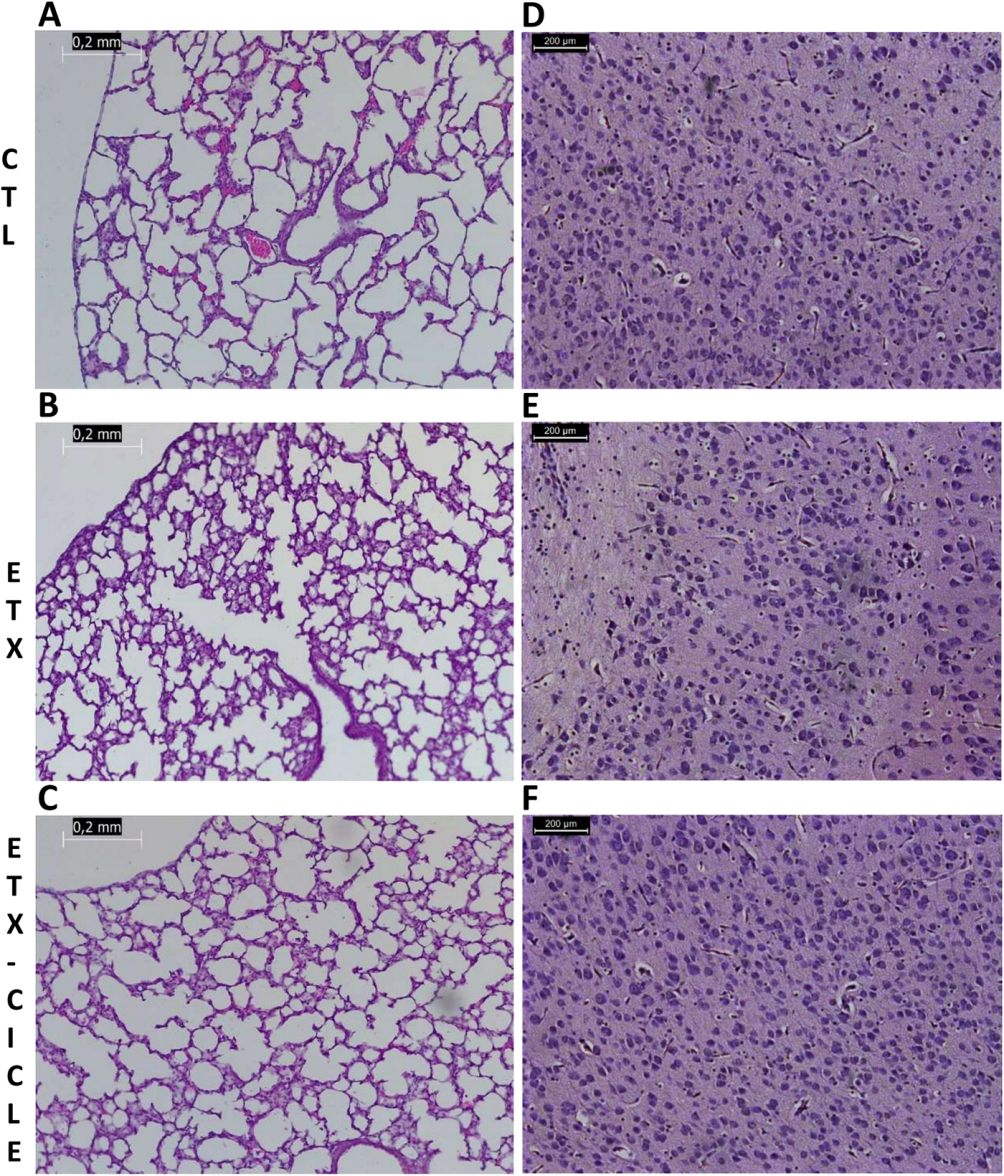
Light microscopic appearance of the lung **(A–C)** and brain **(D–F)** in saline-control rats (CTL), rats who received i.a. exposure to enterotoxin (ETX), and rats who received i.a. exposure to enterotoxin and subcutaneous administration of ciclesonide (ETX-Cicle). The bar represents 0.2 mm/200 µm.

At P14, lung weight (Table 1) was similar across all the groups evaluated. In lung morphometric analysis, the ETX group showed less alveolarization (RAC) (Figure 2A) and lower PVD (Figure 2B), together with higher MLI (Figure 2C) and PVWT (Figure 2D) values than in the CTL group. Ciclesonide treatment (ETX-Cicle group) was associated with recovery RAC (Figure 2A), MLI (Figure 2C), and PVWT (Figure 2D) values in comparison with those in the ETX group (Figures 3A–C).

### 3.4 Right ventricular hypertrophy

**Figure 4:**
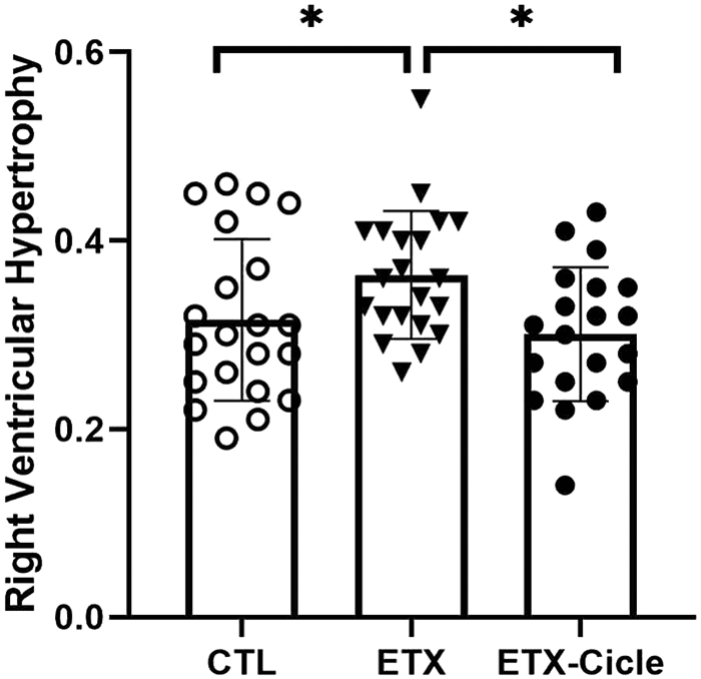
Effect of ciclesonide administration on pulmonary hypertension or right ventricular hypertrophy in an experimental i.a. enterotoxin administration model in saline-control rats (CTL), rats who received i.a. exposure to enterotoxin (ETX), and rats who received i.a. exposure to enterotoxin and subcutaneous administration of ciclesonide (ETX-Cicle). The numbers of pups studied in each group are as follows: CTL, *n* = 21; ETX, *n* = 23; ETX-Cicle, *n* = 20. *p < 0.05 vs. ETX group.

Although heart weight was similar in CTL and both ETX groups at P14 (Table 1), neonatal rats exposed to i.a. enterotoxin without treatment (ETX group) showed more right ventricular hypertrophy than the CTL group at P14 (Figure 4). Neonatal rats exposed to i.a. enterotoxin that received ciclesonide treatment showed significantly less right ventricular hypertrophy than the ETX group at P14, with weight ratios similar to those in the CTL group (Figure 4).

### 3.5 Brain evaluation

At P14, brain weight was similar across all evaluated groups (no differences reaching significance). Moreover, no significant differences were found in apoptosis, edema, inflammation, hemorrhage, or infarction parameters (Table 2 and Figures 3D–F).

**Table 2:** Effect of ciclesonide administration on brain histology parameters in an experimental intra-amniotic enterotoxin administration model.

Group	Necrosis	Edema	Inflammation	Hemorrhage	Infarct
CTL	1.8 ± 0.4	0	0	0.21 ± 0.09	0
ETX	1.6 ± 0.3	0	0	0.30 ± 0.11	0
ETX-Cicle	1.4 ± 0.3	0	0	0.16 ± 0.08	0

The numbers of pups studied in each group are as follows: control (CTL), *n* = 19; enterotoxin (ETX), *n* = 20; ETX-ciclesonide (ETX-Cicle), *n* = 19. No significant differences were observed between groups.

## 4 Discussion

In our study, intrauterine inflammation, induced by a single dose of i.a. enterotoxin in fetal rats at the late canalicular stage of lung development (a stage of lung development similar to that of human premature newborns born at 24–26 weeks of gestation, who are at very high risk of BPD), was associated with right ventricular hypertrophy and sustained abnormalities in infant lung structure, characterized by reduced alveolarization, decreased vessel density, and higher PVWT. Notably, the administration of ciclesonide, an unexplored glucocorticoid in preterm neonates, reverted the effects of intrauterine inflammation on the lung and heart without causing any apparent histological abnormalities in the brain.

The etiology of BPD is multifactorial and may involve antenatal factors such as antenatal inflammation, placental dysfunction, intrauterine infection, and chorioamnionitis, which are known to be linked to causes of preterm labor, preterm birth, BPD, and late respiratory disease during infancy. BPD is a chronic lung disease of infancy that develops following premature birth and injury to the immature lung, and it continues to be one of the most significant sequelae of prematurity despite improvements in perinatal care (1, 2). Any intervention or treatment for BPD prevention could have a major impact on the quality of life, healthcare needs, and costs of these patients throughout their infancy, childhood, and adulthood.

To mimic human BPD, newborn animal lungs may be subjected to various stimuli such as hyperoxia, prolonged mechanical ventilation, or infection/inflammation (22). There is growing evidence supporting that an important role is played by antenatal factors, such as exposure to chorioamnionitis, which is among the major determinants of BPD, pulmonary hypertension, and late respiratory disease in preterm infants (23). Although classical preclinical models of BPD have been based on exposure to hyperoxia (24), many studies using a preterm pig model (16) or a rat model, similar to the one we have used (17, 18, 25, 26), have demonstrated that intrauterine inflammation (using a single dose of i.a. ETX) may be sufficient to induce prolonged impairment of alveolarization and vascular growth characteristic of BPD. Such models may help improve our understanding of the pathogenesis of neonatal pulmonary hypertension.

In our study, neonatal rats at P14 previously given an i.a. ETX injection developed significant impairments in lung mechanics compared with neonatal rats given an i.a. saline injection. Moreover, the ETX group also showed altered lung structure (sustained abnormalities in alveolar and vascular growth) combined with signs of pulmonary hypertension (greater right ventricular hypertrophy), which seems to mimic features of human BPD (17).

After chorioamnionitis, a simplification in alveolar and microvascular structure has been described, induced by a cascade of lung injury, pulmonary inflammation, and remodeling of the fetal lung (27). The persistence and non-resolution of lung inflammation leads to BPD. Antenatal glucocorticoids are frequently given in pregnancies complicated by chorioamnionitis and are used postnatally to prevent and treat BPD (28). In particular, dexamethasone and hydrocortisone have been used due to their potent anti-inflammatory effects (inhibiting the synthesis of proinflammatory mediators, including macrophages, eosinophils, and lymphocytes, and suppressing phospholipase A2, which is responsible for the production of numerous inflammatory mediators) (29). On the other hand, budesonide has strong local anti-inflammatory effects with fewer systemic adverse reactions compared to other glucocorticoids. When administered in combination with pulmonary surfactant, it has been shown to effectively shorten hospital stay, reduce the duration of invasive mechanical ventilation, and decrease the incidence of BPD (29). However, in this case, budesonide administration is conditioned to surfactant administration, which is not required in all cases. Nonetheless, other candidate drugs should be considered for use in BPD to avoid the short- and long-term adverse effects (gastrointestinal bleeding, poor growth, neurodevelopmental impairment, and adrenal insufficiency, among others) of dexamethasone and/or hydrocortisone (12, 13) or the need of surfactant administration (29).

Ciclesonide is a synthetic inhaled glucocorticoid approved for asthma treatment with few systemic side effects (30). It is a prodrug converted by carboxylesterases enriched in the lower airway into the active compound des-ciclesonide, which has a 100-fold higher binding affinity to the glucocorticoid receptor than the inactive compound (14). Compared with other systemic or inhaled glucocorticoids, des-ciclesonide has optimal pharmacokinetics, with 99% of the drug being protein-bound in the serum (bioavailability of <1%) and >99% undergoing first-pass metabolism in the liver, making it an ideal candidate for avoiding systemic and neurologic effects (14). Nonetheless, this drug has not previously been used to treat neonatal lung diseases such as BPD. In our study, the postnatal administration of ciclesonide for 5 days to neonatal rats exposed to i.a. enterotoxin was associated with significant improvements in lung function (PIP and compliance values) and lung structure (RAC, MLI, and PVWT), as well as a reversal of right ventricular hypertrophy compared to the group exposed only to ETX; these benefits were observed during the rodent neonatal period (P14) without any apparent effects on neonatal brain development. The positive effects without adverse effects in our model could be explained by its optimal pharmacokinetics, activation in the lower airway, and high first-pass metabolism in the liver, as described in a neonatal healthy rat model (14). In the Jaumotte et al. study (14), neonatal pups received either a vehicle, dexamethasone, or ciclesonide (subcutaneous, 0.5 mg/kg from PN1–PN5); although this study was performed using healthy neonatal rats (not using a BPD-like model), the administration of ciclesonide was able to achieve better outcomes than dexamethasone (which was associated with skin and fur abnormalities, somatic growth restriction, and decreased brain weight and white matter development).

In conclusion, to date, the effective management and prevention of BPD continues to be a challenge; therefore, our promising results using ciclesonide in an animal model that mimics human BPD opens a window of hope. In particular, the use of this new drug was associated with a positive impact on lung function and structure, with limited adverse extra-pulmonary effects—notably, no repercussions for the developing brain. However, we recognize the need to bridge the gaps between this animal model and its application in humans. Given our initial promising data, more long-term studies (extending into adulthood), prolonged ciclesonide treatment (>5 days), and studies comparing ciclesonide vs. other glucocorticoid term treatments (in a BPD-like model) could be useful.

## Data Availability

The raw data supporting the conclusions of this article will be made available by the authors without undue reservation.
